# Changing Dynamics of Human–Rickettsial Interactions

**DOI:** 10.4269/ajtmh.15-0762

**Published:** 2016-01-06

**Authors:** David H. Walker

**Affiliations:** Department of Pathology and Center for Biodefense and Emerging Infectious Diseases, University of Texas Medical Branch at Galveston, Galveston, Texas

Fluctuations in the reported incidence of scrub typhus, Rocky Mountain spotted fever (RMSF), and Mediterranean spotted fever have been noted over the past century since their recognition as arthropod-borne infections, with decade-long periods between the peaks and troughs. In Japan, classic *Leptotrombidium akamushi*-transmitted scrub typhus occurred with a high incidence before the 1950s, and thereafter decreased dramatically, essentially disappearing by the early 1980s, at which time a tremendous rise in scrub typhus transmitted by *Leptotrombidium pallidium* and *Leptotrombidium scutellare* mites occurred.^1^ The report by Jeung and others in this issue^2^ addresses scrub typhus in South Korea, where the first case was diagnosed in 1985 and a rise to 10,365 cases was reported in 2013. By studying the times of peak incidence in provinces representing northern, central, and southern Korea over a 13-year-period during which the number of cases increased tremendously, they noted that the peak occurred progressively later in the year. Increase in the density of *L. scutellare*, the vector of *Orientia tsutsugamushi*, in northern and central Korea correlated with the increased incidence of disease. The possibility of global climate change as a factor contributing to these changes is supported by the effect of longer seasonal warm temperatures and *L. scutellare* abundance in temperate regions of Korea.^3^

Trombiculid mites feed particularly on rodent hosts such as *Rattus* species, and no longer remain active when the ground freezes. Because *O. tsutsugamushi* is apparently maintained solely by transovarian transmission in mites and is transmitted by larvae (the only stage of this arthropod that feeds on a host), the occurrence of scrub typhus depends on the abundance of infected chiggers and their contact with humans. The appearance of scrub typhus in recent years in areas where the illness was nearly forgotten, such as in India, Sri Lanka, the Maldives, and Micronesia, and in regions where it had not been known previously such as north of the Yangtze river in China and in Korea suggests that environmental changes including potential northward spread of infected mites may be occurring.^4^–^6^ Increased diagnosis of scrub typhus in Thailand, Taiwan, Japan, and Laos may represent truly increased incidence, greater awareness, or greater availability of diagnostic methods.

Whatever the explanation for increasing incidence and geographic range, scrub typhus begs for greater medical, public health, and scientific attention. Diagnosis is difficult, frequently missed, and would benefit greatly from a low-cost point-of-care diagnostic tool that is effective early in the illness, when therapeutic decisions are being made. With an estimated 1 million cases annually and an average case-fatality rate of 10%, a vaccine that provides cross protection against 70 or more strains of *O. tsutsugamushi* is a true public health need.^7^ Research should begin with determination of protective immune mechanisms and with genome-wide bioinformatic analyses of potential protective antigens. Currently, excellent research on the pathobiology of *O. tsutsugamushi*–host interactions is being accomplished in Korea, but the development of an effective vaccine is far from near.

Articles from the Centers for Disease Control and Prevention (CDC) in this issue describe a tremendous increase in reported cases of spotted fever group rickettsioses (SFGR) and the dramatic spread of *Amblyomma americanum* (the lone star tick) in the United States. At first impression, there appears to have been an enormous increase in cases of SFGR during the period 2008–2012. However, it should not be interpreted that these were all patients with RMSF, because the approach most widely used for laboratory confirmation of this diagnosis was detection of antibodies reactive with *Rickettsia rickettsii*, which also cross-react with other SFG rickettsiae including *Rickettsia amblyommii*, *Rickettsia parkeri*, *Rickettsia* strain 364D, *Rickettsia akari*, *Rickettsia massiliae*, and *Rickettsia montanensis*.^8^ As the authors acknowledge, the criteria for a “probable” case are problematic. Indeed, only 1% of cases reported on case report forms in 2008–2014 were confirmed by seroconversion, a 4-fold rise in antibody titer, immunohistochemical detection of SFG rickettsiae, polymerase chain reaction (PCR), or isolation of rickettsiae. Only the latter two methods are specific for a particular *Rickettsia* species; 23 cases of RMSF were identified by PCR and four by culture over the 5-year period. The diagnosis of the remainder of cases was based on a single, often low titer of antibodies. Moreover, the median day of collection of the single serum assayed was day 4 of illness, and in RMSF, antibodies to rickettsiae do not appear until days 7–10 of illness. It should be interpreted that seropositive patients commonly had antibodies preexisting during their acute illness, that infection with ehrlichiae or a virus was a more likely cause of acute illness than rickettsiae, and, thus, that these were unlikely to be SFGR cases. In fact, the clinical case definition for SFGR is a febrile patient who had one or more of the following: rash, eschar, headache, myalgia, anemia, thrombocytopenia, and elevated serum hepatic transaminases. Four of these are also characteristic of human monocytotropic ehrlichiosis.

The case fatality rate of RMSF in the antibiotic era has been 4% or worse.^9^ The case fatality rate in this report of SFGR of 0.4% emphasizes the likelihood that most patients' antibodies were stimulated by an agent other than *R. rickettsii*. Although human infections with *R. parkeri* and *Rickettsia* strain 364D are important discoveries, they are unlikely to account for a substantial portion of the reported cases, as only 3% of persons for whom eschar status is stated were reported to have the tick-inoculation site lesions associated with these rickettsiae.^10^,^11^

The article by Dahlgren and others^12^ suggests the hypothesis that “less pathogenic rickettsiae are causing human infections,” and they document the geographic spread of the lone star tick, which is frequently infected with *R. amblyommii*, a SFG *Rickettsia* of unknown pathogenicity. Indeed, *A. americanum* is the predominant tick in the region where most of the cases of SFGR were reported (e.g., 92–93% of ticks in North Carolina and Arkansas). A large proportion of lone star ticks carry *R. amblyommii* (57% in North Carolina, 56–67% in Virginia, and 88% in Georgia). Strong circumstantial evidence supports *R. amblyommii* as the agent causing seroconversion among soldiers at Fort Chaffee, AR. Soldiers who developed antibodies to SFGR were usually (54–73%) asymptomatic, but were ill significantly more frequently than those without antibodies.^13^,^14^ The soldiers with antibodies more frequently reported headache, myalgia, rash, arthralgia, fever, chills, dyspnea, and confusion than similarly exposed non-seroconverters. Guinea pigs infected with *R. amblyommii* develop a high antibody titer that is cross-reactive with *R. rickettsii* with mild or no signs of illness. When subsequently challenged with a lethal dose of *R. rickettsii*, these guinea pigs are immune.^15^,^16^ If the immunity of guinea pigs could be extrapolated to humans, those previously infected with *R. amblyommii* may actually be protected against RMSF.

The article by Drexler and others^17^ reported the highest case fatality rate (1.6%) in children less than 10 years of age. Historically rickettsial diseases have been less severe at younger ages (in preantibiotic era case fatality rates: 7.6%, under 16 years of age and 25%, persons 16 years of age or older and in 1970–1974: 5.2%, less than 15 years of age; 5.7%, 15–29 years of age; 11.4%, 30–34 years of age; 12.9%, 45–59 years of age; and 19.0%, 60 years or greater of age).^9^,^18^ Moreover, anti-SFG rickettsial antibodies have been reported in 10% or more of children in the lone star tick belt.^19^ Because seroprevalence of persons exposed to pathogens over a lifetime usually shows age-dependent increases, it is likely that fewer children had preexisting antibodies, and thus children with antibodies were more likely to have RMSF, explaining their higher case fatality rate. The apparent increasing incidence with older ages is probably largely based on the preexisting antibodies, and is likely a result of age-dependent seroprevalence.

The investigators at CDC drew important conclusions from these studies: the need for better laboratory methods, documentation and reporting of specific clinical observations, greater clinical awareness, improved utilization of appropriate diagnostic tests including convalescent serology, and reporting of cases. Attention to these recommendations will enable an improved understanding of the epidemiology of RMSF and other rickettsial diseases.
